# Genetic Diversity and Population Structure of Sorghum [*Sorghum Bicolor* (L.) Moench] Accessions as Revealed by Single Nucleotide Polymorphism Markers

**DOI:** 10.3389/fpls.2021.799482

**Published:** 2022-01-05

**Authors:** Muluken Enyew, Tileye Feyissa, Anders S. Carlsson, Kassahun Tesfaye, Cecilia Hammenhag, Mulatu Geleta

**Affiliations:** ^1^Institute of Biotechnology, Addis Ababa University, Addis Ababa, Ethiopia; ^2^Department of Plant Breeding, Swedish University of Agricultural Sciences, Lomma, Sweden; ^3^Ethiopian Biotechnology Institute, Addis Ababa, Ethiopia

**Keywords:** agro-ecological zone, genetic differentiation, geographical region, population structure, sorghum [*Sorghum bicolor* (L.) Moench]

## Abstract

Ethiopia is the center of origin for sorghum [*Sorghum bicolor* (L.) Moench], where the distinct agro-ecological zones significantly contributed to the genetic diversity of the crops. A large number of sorghum landrace accessions have been conserved *ex situ*. Molecular characterization of this diverse germplasm can contribute to its efficient conservation and utilization in the breeding programs. This study aimed to investigate the genetic diversity of Ethiopian sorghum using gene-based single nucleotide polymorphism (SNP) markers. In total, 359 individuals representing 24 landrace accessions were genotyped using 3,001 SNP markers. The SNP markers had moderately high polymorphism information content (PIC = 0.24) and gene diversity (H = 0.29), on average. This study revealed 48 SNP loci that were significantly deviated from Hardy–Weinberg equilibrium with excess heterozygosity and 13 loci presumed to be under selection (*P* < 0.01). The analysis of molecular variance (AMOVA) determined that 35.5% of the total variation occurred within and 64.5% among the accessions. Similarly, significant differentiations were observed among geographic regions and peduncle shape-based groups. In the latter case, accessions with bent peduncles had higher genetic variation than those with erect peduncles. More alleles that are private were found in the eastern region than in the other regions of the country, suggesting a good *in situ* conservation status in the east. Cluster, principal coordinates (PCoA), and STRUCTURE analyses revealed distinct accession clusters. Hence, crossbreeding genotypes from different clusters and evaluating their progenies for desirable traits is advantageous. The exceptionally high heterozygosity observed in accession *SB4* and *SB21* from the western geographic region is an intriguing finding of this study, which merits further investigation.

## Introduction

Sorghum [*Sorghum bicolor* (L.) Moench] is the fifth most important cereal crop in the world next to maize, rice, wheat, and barley in terms of both production and harvested area (FAOSTAT, 2019). It is a major food crop for more than 500 million people across Africa, Asia, and Latin America, particularly for those in the semi-arid tropical regions (Ejeta, 2005). It is grown in drought-prone areas where several other crops cannot reliably grow. Recent FAOSTAT data on annual global production of sorghum showed that it covered about 40 million ha of land and produced grains of ca 57.9 million metric tons (MMT) (FAOSTAT, 2019). The United States, Nigeria, and Ethiopia are the leading sorghum-producing countries in the world with a total production of 8.6, 6.7, and 5.2 MMT, respectively (Statista, 2020). In Africa, sorghum is the second most widely cultivated cereal crop, only surpassed by maize (FAOSTAT, 2019).

Ethiopia is considered as one of the centers of origin and diversity of sorghum (De Wet and Harlan, 1971) due to the presence of wild relatives and diversified forms of the crop in the country. The sorghum gene pool in the country has been used as novel sources of germplasm for crop improvements. For example, genotypes harboring genes that confer resistance to ergot and green bug (Wu et al., 2006) as well as high lysine (Singh and Axtell, 1973) and drought-tolerant (Borrell et al., 2000) sorghum genotypes were identified from the Ethiopian accessions.

Studying the genetic diversity of a crop is very important for effective germplasm management, utilization, and genotype selection for crop improvement (Bucheyeki et al., 2009). It is the most important step for conserving and increasing the rate of genetic gain in crop-breeding programs. The level of genetic diversity within a species is commonly used to measure the level of species adaptability and survival in unpredictable environmental conditions (Rao and Hodgkin, 2002; Govindaraj et al., 2015). Similarly, the level of genetic variation within a population is the basis for germplasm selection in plant breeding and is vital for crop improvement (Mohammadi and Prasanna, 2003). Hence, the conservation and utilization of plant genetic variation are crucial to human food security (Rao and Hodgkin, 2002).

Sorghum is a predominantly self-pollinated diploid species (Poehlman and Sleper, 1979) with 2*n* = 2× = 20 chromosomes. It has a small genome relative to other cereal crops, which is about 730 Mbp (Paterson et al., 2009). Its whole genome was sequenced and made accessible for public use^1^ (Paterson et al., 2009; McCormick et al., 2018), which facilitated the development of DNA markers, such as single nucleotide polymorphism (SNPs) for various applications, including analyses of population genetics and identification of genomic regions associated with complex traits through quantitative trait loci (QTL) and association mapping (Too et al., 2018; Girma et al., 2019).

The genetic diversity of crop species can be studied through morphological, biochemical, and molecular markers (Rao et al., 1996; Geleta and Labuschagne, 2005; Mehmood et al., 2008; Enyew et al., 2021). Previous studies on the genetic diversity of sorghum have been carried out by using random amplified polymorphism DNA (RAPD) analysis (Ayana et al., 2000; Ruiz-Chután et al., 2019), simple sequence repeat (SSR) markers (Djè et al., 2000; Ghebru et al., 2002; Manzelli et al., 2007; Ali et al., 2008; Wang et al., 2009; Ng’uni et al., 2011, 2012; Burow et al., 2012; Adugna et al., 2013; Adugna, 2014; Mofokeng et al., 2014; Motlhaodi et al., 2014, 2017), and express sequence tags (EST) SSR markers (Ramu et al., 2013), SNP markers (Cuevas et al., 2017; Afolayan et al., 2019; Cuevas and Prom, 2020). More recently, a few studies have been performed on the genetic diversity of Ethiopian sorghum accessions using SNP markers (Girma et al., 2019; Menamo et al., 2021; Wondimu et al., 2021). These studies brought out the contribution of geographic regions and agro-ecological zones for the genetic variation and population structure of sorghum grown in Ethiopia. However, these studies did not consider genetic variation within populations, as the analyses were based on either a single plant per accession or a pool of individual plants treated as a single sample per accession). Ethiopian Biodiversity Institute (EBI) has conserved more than 9,432 sorghum accessions collected from diverse agro-ecologies across the country.^2^ However, the genetic diversity of most of the accessions in the collection remains molecularly uncharacterized. Therefore, this study analyzes the genetic diversity and population structure of selected Ethiopian sorghum accessions using SNP markers in order to generate highly important information, which together with previous research results, lead to deeper insight on the sorghum gene pool in the country and beyond.

## Materials and Methods

### Plant Materials

Twenty-four Ethiopian sorghum landrace accessions originally collected by the EBI were obtained from Melkassa Agricultural Research Center (MARC). The accessions were selected to represent three agro-ecological zones according to the classification by Amede et al. (2015) viz. cool/subhumid, cool/semiarid, and warm/semiarid zones (Supplementary Figure 1). Supplementary Table 1 provides details about these accessions, including the sampling locations, as well as major morphological and phenological characteristics. Photographs showing panicle diversity in the Ethiopian sorghum that represents these accessions are provided as Supplementary Figure 2.

### Planting, Sampling, and Genomic DNA Extraction

Sorghum seeds representing the 24 accessions were planted using plastic pots filled with soil in a greenhouse at the Department of Plant Breeding, SLU, Sweden. Two weeks after planting, the leaf tissues from individual plants were collected using a sample collection kit provided by LGC-Genomics (Berlin, Germany), as described by Tsehay et al. (2020). Each accession was represented by 15 individual plants, except accession *SB10*, which was represented by 14 individuals; hence 359 genotypes were sampled in total. The samples were then sent to LGC Genomics (Berlin, Germany) where genomic DNA extraction was conducted for subsequent genotyping. High-quality genomic DNA, suitable for next-generation sequencing (NGS), was extracted using the Sbeadex plant kit.^3^

### SNP Selection, Assay Design, Sequencing, and Genotype Calling

The vast majority of SNPs (97%) used in this study were selected from sorghum genome SNP database SorGSD,^4^ a web-portal that provides genome-wide SNP markers for diverse sorghum genetic resources (Luo et al., 2016). Among different sorghum lines in the database, *Cherekit* (an Ethiopian sorghum landrace accession) was targeted for selecting the SNPs. For genotyping, SeqSNP method (an advanced NGS method for genotyping target SNPs) was used. Initially, 12,316 SNPs were targeted for high-specificity (without allowing for off-target hit) assay design, using *Sorghum bicolor* v3.1.1 genome in Phytozome 12.1^5^ as a reference (Paterson et al., 2009; McCormick et al., 2018). Additionally, 380 SNPs within functionally annotated sorghum genes were identified through the Basic Local Alignment Search Tool (BLAST), searching the genes targeting *S. bicolor* v3.1.1 genome sequence using Phytozome 12.1 search function were targeted for the assay design. Out of the total 12,696 targets used for the high specificity assay design, 9,495 were totally covered (two oligo probes per target), 1,631 were partially covered (one oligo probe), whereas 1,190 failed.

For the seqSNP genotyping, 5,000 SNPs were selected among the totally covered SNPs, based on their distribution across the sorghum genome. The number of SNPs targeted on chromosome-1 to chromosome-10 included in the order, 532, 521, 572, 506, 497, 515, 437, 446, 465, and 509 (refer to Supplementary Table 2). One hundred fifty-seven of these SNPs belong to 51 functionally annotated genes (Supplementary Table 3). This was followed by the construction of SeqSNP kit LGC, Biosearch Technologies (Berlin, Germany) comprising 10,000 high-specificity oligo probes for the 5,000 target SNPs and construction of a sequencing library. The target sequencing was conducted using Illumina NextSeq 500/550 v2 system with 75 bp single read sequencing mode. In the end, ca 973,000 reads per sample were obtained and the effective target of SNP coverage per sample was 175 times on average. After sequencing, the reads were adapter-clipped and quality-trimmed to get a minimum Phred quality score of 30 over a window of ten bases. After discarding reads shorter than 65 bases. the quality trimmed reads were aligned against the reference genome using Bowtie2 v2.2.3 (Langmead and Salzberg, 2012), and the SNP genotyping pipeline was set to diploid genotyping with a minimum coverage of eight reads per sample per locus. The variant identification and genotype calling were done using Freebayes v1.0.2-16 (Garrison and Marth, 2012).

### Data Analysis

The site frequency spectra were analyzed for each accession using DnaSP version 6 (Rozas et al., 2003). The nucleotide diversity (Nei, 1987) and Tajima’s D (Tajima, 1989) were calculated using the PopGenome package (Pfeifer et al., 2014) in R software (R Core Team) to reveal the genome-wide pattern of variation using a sliding window approach (window size = 1 Mb, step size = 200 kb), in line with previous studies in sorghum (Yan et al., 2018), maize, and common bean (Lai et al., 2010; Cortés and Blair, 2018).

The mean effective number of alleles (Ne), observed heterozygosity (Ho), expected heterozygosity (He), Shannon information index (I), and gene flow (Nm) for each SNP marker and accession were estimated using GenAlEx 6.5 (Peakall and Smouse, 2012), and the gene diversity (H) and the polymorphism information content (PIC) were performed using PowerMarker (Liu and Muse, 2005). The Hardy–Weinberg equilibrium (HWE) test was also done using GenAlEx 6.5.

Analysis of Molecular Variance (AMOVA) within and among the accessions as well as at higher hierarchical levels were done using the software, Arlequin ver. 3.5.2.2 (Excoffier and Lischer, 2010). Arlequin was also used for estimating pairwise genetic differentiation between accessions and groups and for detecting outlier SNP markers through a non-hierarchical finite island model. The significance of the differentiation of accessions and groups was tested by 10,000 permutations. The joint distribution of population differentiation (F_ST_) and heterozygosity (heterozygosity within populations)/(1 – F_ST_) were obtained according to Excoffier and Lischer (2010). The loci under selection were identified based on the F_ST_ significance level of *P* < 0.01.

The principal coordinates analysis (PCoA) was done using GenAlEx 6.5. The bootstrap-supported unweighted pair group method with arithmetic mean (UPGMA) clustering based on Nei’s genetic distance (Nei and Takezaki, 1983) was performed using PowerMarker v 3.25 (Liu and Muse, 2005) and the resulting trees were visualized using MEGA-X (Kumar et al., 2018). STRUCTURE v. 2.3.4 software (Pritchard et al., 2000) was used for the Bayesian clustering of the 359 individuals representing the 24 sorghum accessions, at the burn-in period length of 100,000 and a Markov Chain Monte Carlo (MCMC) replications of 100,000. The structure analysis was done for K ranging from two to ten, with ten iterations at each K, to determine the optimum number of clusters (genetic populations). The optimum K value was predicted following the simulation method of Evanno et al. (2005) using STRUCTURE HARVESTER version 0.6.92 (Earl, 2012). A bar plot for the optimum K was determined through Clumpak beta version (Kopelman et al., 2015).

## Results

### The Quality and Level of Polymorphism of SNP Markers

In the data matrix of 5,000 SNP loci for the 359 sorghum genotypes, missing data accounted for 2.8% and, of those with data, 94.1% were homozygous. Among the 5,000 target SNP loci, 4,301 (86%) were polymorphic, whereas 699 loci (14%) were monomorphic across the 359 genotypes. Among the 4,301 SNP loci, 4,256, 42, and 1 were bi-, tri- and tetra-allelic, respectively, whereas two loci had the combination of SNP and length variants. Tri- and tetra-allelic loci were excluded from further analysis. The filtering of the 4,256 bi-allelic SNP data, based on the percentage of missing data and minimum allele frequency (MAF) resulted in different numbers of loci varying from 2,259 loci with less than1% missing and greater than10% MAF to 3,089 loci with less than 5% missing and greater than 5% MAF. For the population genetics analyses, 3,001 bi-allelic SNP loci with missing data less than 2% and MAF greater than 5% were used (Supplementary Figure 3). About 26% of the markers had MAF between 5 and 10% whereas 31% had MAF between 11 and 20%. About 40% of the markers had MAF greater than 2% (Supplementary Figure 3 and Supplementary Table 4). The SNP markers had a moderately balanced distribution across the chromosomes, ranging from 252 SNPs (8.4%) on chromosome 8–371 SNPs (12.4%) on chromosome 3 (Supplementary Table 4).

**FIGURE 1:**
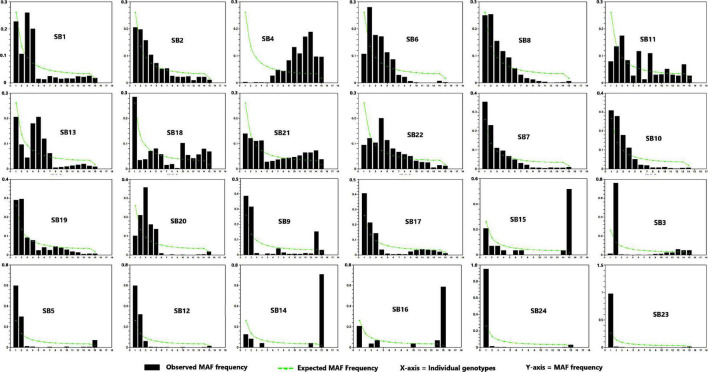
The pattern of site frequency spectrum based on the proportion of the minor allele frequency (MAF) of single nucleotide polymorphism (SNP) in the 24 sorghum accessions.

**FIGURE 2:**
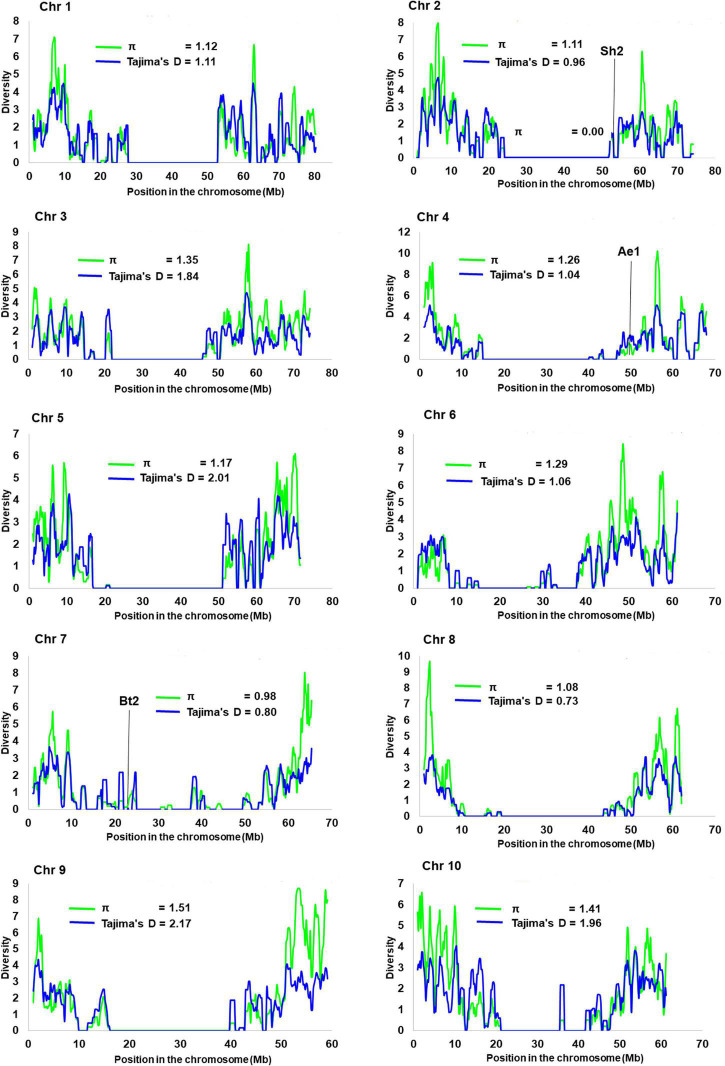
Genome-wide pattern of diversity in the 359 individual plants representing the 24 sorghum accessions. A sliding window approach (window size = 1 Mb, step size = 200 kb) was used to analyze nucleotide diversity and Tajima’s D. The black vertical lines on chromosomes 2, 4, and 7 show the positions of *shrunken2* (sh2), *amylose extender1* (ae1), and *brittle2* (bt2), respectively, which were previously identified as domestication loci in maize and sorghum that are localized at regions of low diversity. The overall average nucleotide diversity (π) and Tajima’s D were 1.2/Mb and 1.4/Mb, respectively.

**FIGURE 3:**
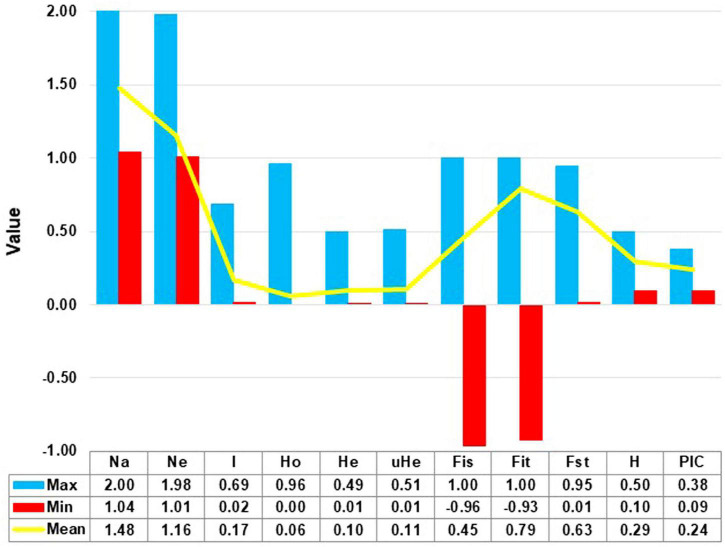
The mean, minimum (Min), and maximum (Max) values for the number of allele (Na), number of effective allele (Ne), Shannon informative index (I), observed heterozygosity (Ho), unbiased expected heterozygosity (uHe), expected heterozygosity (He), fixation indices (Fis, Fit, Fst), polymorphic information content (PIC), and gene diversity (H) for the 3001 polymorphic SNP loci.

The site frequency spectrum revealed high variation in the MAF distribution of the SNPs among the 24 sorghum landrace accessions (Figure 1). All individuals in 67% of the accessions (horizontally, the first 16 accessions in Figure 1) had major alleles in most of their SNP loci although to a different extent. Interestingly, one individual from each of the remaining eight accessions carried minor alleles across most of their SNP loci. The expected site-frequency spectrum determined using a coalescent approach matched the observed frequency distributions fairly well for the accessions, *SB2*, *SB7*, and *SB19* while it was inversely related to the observed frequency distributionsin accession *SB4* (Figure 1). The genome-wide diversity across sorghum landrace accessions was quantified using a sliding window approach (window size = 1 Mbp, step size = 200 kb) to explore the genomic signatures of diversity in sorghum. The analyses resulted in an overall average nucleotide diversity (π) and Tajima’s D of 1.2 and 1.4/Mb, respectively (Figure 2). It is clear from Figure 2 that SNPs representing the centromere regions of each chromosome almost do not exist among the 3,001 SNPs used in this study, and thus the diversity estimates were extremely low or zero. At chromosome level, the highest average nucleotide diversity and Tajima’s D were recorded in chromosome 9 (1.5 and 2.2/Mb) and the lowest in chromosome 7 (1.0/Mb) and chromosome 8 (0.7/Mb), respectively (Figure 2). Previously reported candidate loci for domestication are found at the genomic regions with notably low diversity on chromosomes 2, 4, and 7 (Figure 2). The effective number of alleles found across the 3,001 SNP markers ranged from 1.01 to 1.98 with a mean of 1.16. Observed heterozygosity (Ho) varied from 0.0 to 0.96 with a mean of 0.06 while the mean expected heterozygosity (He) was 0.10 with individual values per locus ranging from 0.01 to 0.49. Similarly, the gene diversity estimates per locus varied from 0.10 to 0.50 with a mean value of 0.29 (Figure 3 and Supplementary Table 4). The average PIC of the loci was 0.24 with individual values ranging from 0.09 to 0.37. In the case of fixation indices, the minimum, maximum, and mean values for F_IS_ were –0.96, 1.00, and 0.45, for F_IT_ they were –0.93, 1.00, and 0.79, and for F_ST_, they were 0.01, 0.95, and 0.63, respectively. The estimates of gene flow (Nm) per locus showed wide variation, ranging from 0.01 to 20.23, with a mean of 0.20 (Figure 3 and Supplementary Table 4).

Based on the HWE test, 99.5% of the SNP markers showed significant deviation from HWE (Supplementary Table 4). Among the 3,001 SNP loci, 97.9% were heterozygote-deficient, whereas 1.6% (48 loci) had excess heterozygosity showing significant deviation from HWE (*P* < 0.05). The candidate genes containing SNP markers showing excess heterozygosity and their annotated functions were retrieved from SorGSD^6^ and further evaluated. Among the 48 SNP loci that showed excess heterozygosity, nine SNPs lacked one of the three possible genotypes expected in a bi-allelic polymorphic locus under the assumption of HWE. The change in amino acid sequences of the corresponding genes was obtained in all SNPs, except three SNPs (snp_sb001000020838, snp_sb042060612417, and snp_sb042061102446) (Supplementary Table 5).

### Genetic Diversity Analysis

The 3,001 polymorphic SNP markers revealed a wide range of variation in the Ethiopian sorghum germplasm, as estimated using different population genetics parameters across the 24 accessions, and are summarized in Table 1. The effective number of alleles of the accessions varied from 1.01 to 1.46 with a mean of 1.21, whereas the mean Shannon’s Information index (I) was 0.25 with individual values ranging from 0.0 (*SB14* and *SB15*) to 0.42 (*SB21*). The lowest and the highest Ho values varied from 0.01 (*SB5*, *SB14*, and *SB15* and *SB16*) to 0.25 (*SB21*) with a mean of 0.07. Likewise, the He and unbiased expected heterozygosity (uHe) of the accessions ranged from 0.0 to 0.27 and 0.0 to 0.28, respectively with a mean of 0.15 (Table 1). The lowest values were recorded in accessions, *SB14, SB15*, and *SB16*, whereas *SB21* recorded the highest values for these parameters. The percent polymorphic loci (PPL) of the accessions varied from 0.8 to 91.4% with a mean of 47.7%. The fixation index (F) showed wide variation with values ranging from –0.76 (*SB14*) to 0.84 (*SB3*). Overall, accession *SB21* showed the highest value for Ne, Ho, He, uHe, I, and the number of locally common alleles (NLCA) and PPL while *SB14*, *SB15*, and *SB16* showed the lowest values for all genetic diversity parameters analyzed (Table 1).

**TABLE 1:** Summary of different genetic diversity estimates based on 3,001 SNP markers for each of the 24 sorghum accessions and for a group of accessions grouped according to different agro-ecological zones (cool/semiarid, cool/subhumid, and warm/semiarid), geographical regions (eastern, northern and western), and peduncle shape (bent and erect).

Accession	Na	Ne	I	Ho	He	uHe	*F*	PPL	NPA	NLCA
SB1	1.806	1.248	0.282	0.064	0.169	0.174	0.494	80.6	0.0	0.008
SB2	1.663	1.214	0.239	0.080	0.144	0.149	0.344	66.3	0.0	0.006
SB3	1.317	1.101	0.108	0.023	0.065	0.067	0.837	31.7	0.0	0.003
SB4	1.459	1.394	0.298	0.157	0.210	0.217	0.255	45.9	0.0	0.006
SB5	1.104	1.018	0.023	0.013	0.013	0.013	0.196	10.4	0.0	0.002
SB6	1.832	1.221	0.284	0.059	0.165	0.171	0.607	83.2	0.0	0.008
SB7	1.484	1.110	0.142	0.043	0.081	0.083	0.380	48.4	0.0	0.004
SB8	1.740	1.175	0.229	0.090	0.131	0.135	0.262	74.0	0.0	0.006
SB9	1.292	1.095	0.095	0.019	0.058	0.060	0.503	29.2	0.0	0.003
SB10	1.741	1.161	0.217	0.072	0.122	0.126	0.322	74.1	0.0	0.007
SB11	1.427	1.200	0.192	0.039	0.123	0.128	0.557	42.7	0.0	0.003
SB12	1.433	1.051	0.086	0.031	0.043	0.044	0.242	43.3	0.0	0.005
SB13	1.692	1.235	0.265	0.056	0.162	0.167	0.531	69.2	0.0	0.005
SB14	1.008	1.006	0.005	0.006	0.003	0.003	–0.757	0.8	0.0	0.001
SB15	1.010	1.006	0.005	0.006	0.003	0.003	–0.589	1.0	0.0	0.002
SB16	1.010	1.007	0.005	0.007	0.004	0.004	–0.685	1.0	0.0	0.001
SB17	1.791	1.214	0.239	0.074	0.141	0.146	0.283	79.1	0.0	0.007
SB18	1.504	1.253	0.224	0.065	0.147	0.152	0.368	50.4	0.0	0.003
SB19	1.568	1.153	0.180	0.031	0.106	0.109	0.558	56.8	0.0	0.005
SB20	1.335	1.082	0.110	0.046	0.063	0.065	0.263	33.5	0.0	0.003
SB21	1.914	1.457	0.416	0.253	0.271	0.280	0.038	91.4	0.0	0.008
SB22	1.680	1.271	0.287	0.071	0.179	0.185	0.536	68.0	0.0	0.006
SB23	1.410	1.034	0.063	0.032	0.029	0.030	–0.041	41.0	0.0	0.004
SB24	1.232	1.023	0.038	0.023	0.019	0.019	–0.057	23.2	0.0	0.002
Mean	1.754	1.209	0.250	0.067	0.147	0.152	0.419	47.7	0.0	0.005
cool/semiarid	1.994	1.434	0.419	0.043	0.268	0.269	0.782	99.4	5.0	0.000
cool/subhumid	1.909	1.387	0.381	0.051	0.243	0.244	0.700	90.9	0.0	0.000
warm/semiarid	1.995	1.519	0.482	0.089	0.316	0.318	0.660	99.5	5.0	0.000
Mean	1.966	1.447	0.427	0.061	0.276	0.277	0.715	96.6	3.3	0.000
eastern	1.997	1.378	0.389	0.053	0.243	0.244	0.715	99.7	4.0	0.000
northern	1.844	1.291	0.276	0.023	0.176	0.177	0.678	89.8	1.0	0.000
western	1.779	1.272	0.273	0.045	0.170	0.171	0.628	97.4	2.0	0.000
Mean	1.974	1.647	0.535	0.158	0.364	0.369	0.514	95.6	2.3	0.000
bent	2.000	1.564	0.518	0.080	0.341	0.342	0.760	99.9	139.0	0.000
erect	1.954	1.336	0.323	0.030	0.206	0.206	0.650	95.3	1.0	0.000
Mean	1.977	1.450	0.420	0.050	0.274	0.274	0.710	97.6	70.0	0.000

*Na = Number of different alleles; Ne = effective number of alleles; I = Shannon’s information index; Ho = observed heterozygosity; He = expected heterozygosity; uHe = unbiased expected heterozygosity; F = fixation index; PPL = percent polymorphic loci; NPA = number of private alleles; NLCA = number of locally common alleles found in 25% or fewer accessions or group of accessions.*

**FIGURE 4:**
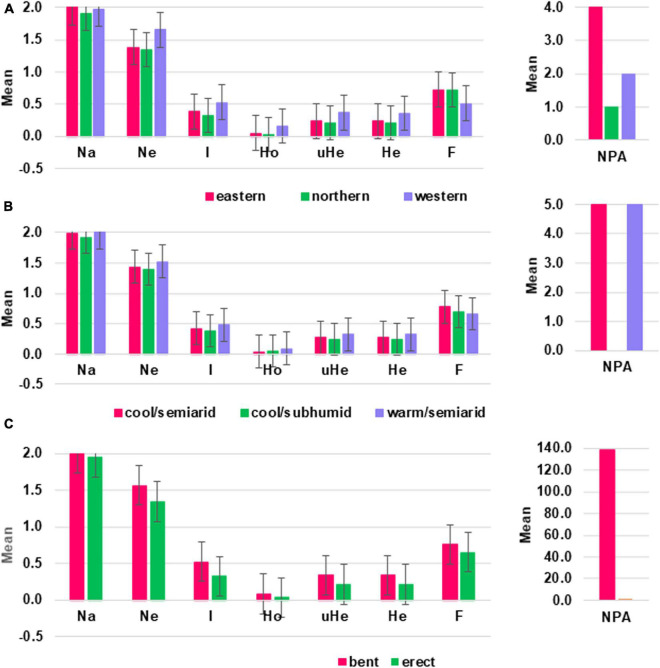
Graphs displaying mean values of different genetic diversity parameters estimated based on 3,001 SNP markers for a group of sorghum accessions grouped according to their **(A)** geographic regions, **(B)** agro-ecological zones, and **(C)** peduncle shape. Na = No. of different alleles; Ne = effective number of alleles; I = Shannon’s information index; Ho = observed heterozygosity; uHe = unbiased expected heterozygosity; He = expected heterozygosity; F = fixation index; NPA = number of private alleles.

Among the agro-ecological zones, warm/semiarid zones showed the highest values for Ne, Ho, He, uHe, and I whereas cool/subhumid zones showed the lowest in the majority of the genetic diversity estimates. Among the groups of accessions in the three agro-ecological zones, the highest value of PPL, which is equal to 99% and the number of private allele per locus were recorded in warm/semiarid and cool/semiarid zones (Table 1 and Supplementary Table 6). In terms of geographic regions, accessions from the western geographic regions showed the highest values in most of the genetic diversity parameters analyzed (I, Ho, He, and uHe) (Figure 4 and Table 1). For example, the eastern, northern, and western accessions had uHe values of 0.24, 0.21, and 0.37, respectively. Accessions from the eastern geographic region showed the highest value PPL, which is equal to 99.7% and in the number of private alleles. Four private alleles were recorded for the eastern region with MAF ranging from 0.19 to 0.47 whereas two and one private alleles were detected in the accessions originated from the western and northern regions, respectively (Table 1 and Supplementary Table 6). With regard to peduncle shape, accessions with bent peduncles were more diverse than those with erect peduncles as shown by the values of I, Ho He, uHe, and PPL (Figure 4 and Table 1). One hundred thirty-nine alleles were specific to accessions with bent peduncles shape with MAF ranging from 0.09 to 0.32, whereas only one private allele with MAF of 0.14 was specific to accessions with erect peduncles (Supplementary Table 6).

### Genetic Differentiation of Accessions and Hierarchical Groups

The results of the AMOVA without grouping the accessions showed that 64.5% of the total variation was observed among accessions and 35.5% within accessions (F_ST_ = 0.65; F_IS_ = 0.47, *P* < 0.001) (Table 2). Additionally, hierarchical AMOVA was conducted by grouping the accessions according to their geographic regions, agro-ecological zones of their collection sites, and their peduncle shape. In this analysis, 19.5% of the total variation was observed among the geographical regions, which is a highly significant differentiation (F_CT_ = 0.20, *P* < 0.001). Similarly, significant differentiation was found among peduncle shape groups with 4.3% of the total variation between them (F_CT_ = 0.04, *P* < 0.05) (Table 2). However, only 1.83% of the total variation accounted for the variation among the agro-ecological zones, which is statistically insignificant (F_CT_ = 0.02 and *P* = 0.17) (Table 2).

**TABLE 2:** Analysis of molecular variance (AMOVA) for 24 accessions without grouping, and by grouping them based on their geographic regions, agro-ecological zones, and peduncle shapes.

Source of variation	DF	SS	Variance components	Percentage of variation	Fixation indices	Probability (*P*) value
Among accessions	23	206414.2	292.1 Va	64.5	F_ST_ = 0.65	Va and F_ST_ < 0.001
Among individuals within accessions	335	79341.1	76.0 Vb	16.8	F_IS_ = 0.47	Vb and F_IS_ < 0.001
Within individuals	359	30426.5	84.8Vc	18.7	F_IT_ = 0.81	Vc and F_IT_ < 0.001
Total	717	316181.8	452.9			
Among geographic regions	2	55111.0	95.5 Va	19.52	F_CT_ = 0.20	Va and F_CT_ < 0.001
Among accessions within geographic regions	21	151303.1	235.6 Vb	48.15	F_*SC*_ = 0.60	Vb and F_*SC*_ < 0.001
within accessions	694	109767.6	158.2 Vc	32.33	F_ST_ = 0.68	Vc and F_ST_ < 0.001
Total	717	316181.8	489.2			
Among agro-ecological zones	2	21393.9	8.3 Va	1.83	F_CT_ = 0.02	Va and F_CT_ = 0.17
Among accessions within agro-ecological zones	21	185020.3	289.3 Vb	63.47	F_*SC*_ = 0.65	Vb and F_*SC*_ < 0.001
within accessions	694	109767.6	158.7 Vc	34.70	F_ST_ = 0.65	Vc and F_ST_ < 0.001
Total	717	316181.8	455.8			
Among peduncle shape groups	1	15762.6	19.9 Va	4.30	F_CT_ = 0.04	Va and F_CT_ < 0.05
Among accessions within peduncle shape groups	22	190651.5	284.4 Vb	61.50	F_*SC*_ = 0.64	Vb and F_*SC*_ < 0.001
Within accessions	694	109767.6	158.17 Vc	34.20	F_ST_ = 0.66	Vc and F_ST_ < 0.001
Total	717	316181.8	462.4			

*The 24 accessions were grouped into four geographic regions, three agro-ecological groups based on world classifications (Amede et al., 2015), and two peduncle shape groups (Supplementary Table 1). PS, peduncle shape; DF, degrees of freedom; SS, sum of square; Va, Vb, and Vc, variance explained by the source of variation; F_ST_, F_IS_, F_IT_, F_CT_ and F_SC_, fixation indices.*

### Population Differentiation and Gene Flow

**FIGURE 5:**
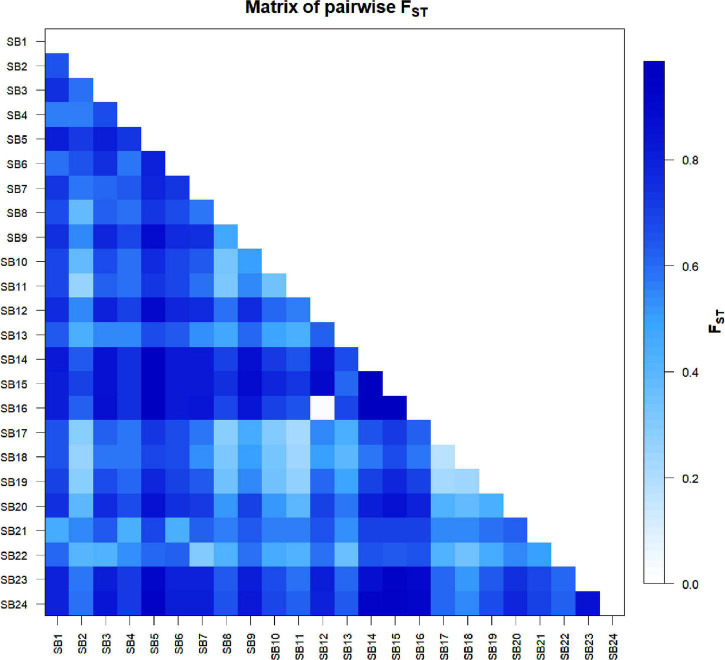
Graphical display of pairwise genetic differentiation (F_ST_) among the 24 sorghum accessions. The differentiation between each pair was significant (*P* < 0.05) except in the case of SB12 vs. SB16.

The pairwise population differentiation analysis revealed significant differentiation among all pairs of accessions with F_ST_ values ranging from 0.18 to 0.99 (Figure 5 and Supplementary Table 7) except in the case of *SB16* vs. *SB12*, which was not significant (F_ST_ = 0.02, *P* > 0.05). The pairs of accessions with the highest F_ST_ value (0.99) were *SB14* vs. *SB15* and *SB15* vs. *SB16*, corresponding to the lowest estimate of gene flow (Nm = 0; Supplementary Table 7). The mean F_ST_ values for the differentiation of each accession from all other accessions varied from 0.47 to 0.81. Accessions *SB15*, *SB14*, and *SB5* were the most differentiated with F_ST_ values of 0.81, 0.80, and 0.78, respectively, whereas *SB18* was the least differentiated accession (F_ST_ = 0.47) (Figure 5 and Supplementary Table 7).

**FIGURE 6:**
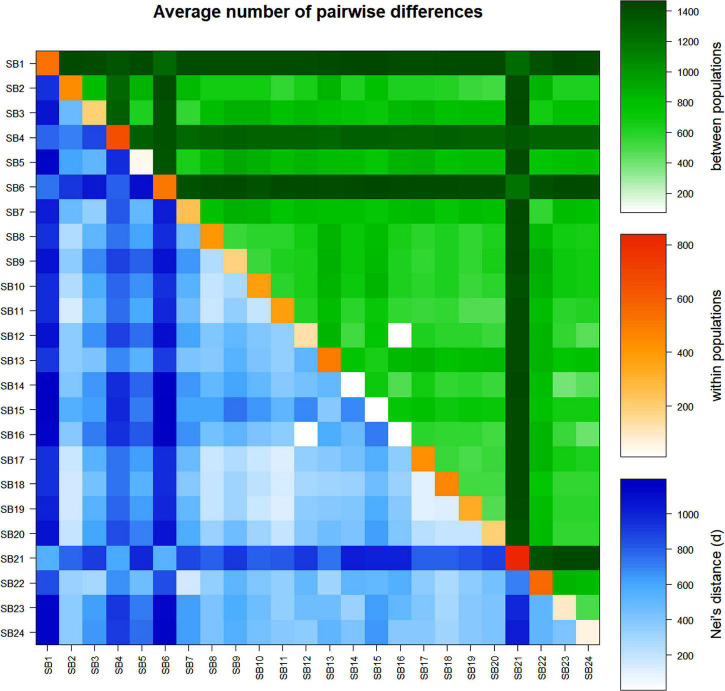
Average number of pairwise differences within and between sorghum accessions: average number of pairwise differences among the accessions (π_xy_) (above diagonal, in green), average number of pairwise differences within accessions (π) (diagonal, in orange), and pairwise net number of allele differences among the accessions (d) (below diagonal, in blue).

The analyses of the average number of pairwise differences (π*_*xy*_*) and the pairwise net number of allele differences (Nei’s distance, *d*) between the accessions revealed a wide variation with π*_*xy*_* ranging from 1.2 (*SB12* vs. *SB16*) to 1,197.2 (*SB6* vs. *SB15*) and *d* ranging from 0.001 (*SB12* v.s *SB16*) to 0.56 (*SB21* vs. *SB6* and SB1 vs. *SB14*) (Figure 6 and Supplementary Table 8). Accessions *SB1*, *SB6*, and *SB21* also showed a higher pairwise net number of allele differences (Nei’s distance, *d*) with other accessions (Figure 6 and Supplementary Table 8). In line with the results of the pairwise F_ST_ analysis, the average number of pairwise differences and Nei’s distance were the lowest for *SB16* vs. *SB12* suggesting that these two accessions are genetically very similar. The average number of pairwise differences within accessions also showed wide variation with the values ranging from 10 (*SB14*) to 840 (*SB21*). This parameter was very low for *SB15* and *SB16*, as with *SB14* (Figure 6 and Supplementary Table 8).

At the geographic region level, the pairwise F_ST_ values among each pair of the three groups were significant (*P* < 0.001). However, accessions from the western region were the most distinct with higher differentiation from those from the northern and eastern regions (F_ST_ = 0.40 and 0.35, respectively). Among the three pairs, accessions from the eastern vs. northern regions were the least differentiated (F_ST_ = 0.12) (Supplementary Table 7). Similar to that of geographic regions, the F_ST_ values among each pair of the accessions from the three agro-ecological zones were also significant (*P* < 0.001). The accessions belonging to the cool/subhumid group were the most differentiated having F_ST_ values of 0.13 and 0.10 against warm/semiarid and cool/semiarid groups, respectively. The warm/semiarid vs. cool/semiarid groups were the least differentiated (F_ST_ = 0.08) among the three pairs (Supplementary Table 7). The average number of pairwise differences and Nei’s distance among the geographic regions and agro-ecological zones had a similar pattern with that of pairwise F_ST_-based differentiation, revealing that the western region was the most differentiated group. In the case of pairwise differences within regions, accessions from the western region had the highest variation whereas the lowest was recorded for the northern region. With regard to agro-ecological zones, warm/semiarid and cool/subhumid zones showed the highest and lowest variations, respectively (Supplementary Table 7).

The non-hierarchical finite island model-based analysis involving the examination of the joint distribution of F_ST_ and heterozygosity among accessions to detect loci under selection revealed 74 SNP loci that were highly significant (*P* < 0.01). Among them, 61 loci had low F_ST_ value (ranging from –0.02 to 0.35), and hence were considered as candidates for balanced selection. Whereas 13 loci (Table 3) had high F_ST_ values (ranging from 0.81 to 0.94), and hence considered as under directional selection. The MAF of these loci ranged from 0.05 to 0.34. The markers were distributed on chromosomes 1, 5, 6, 7, and 9 with over 50% of them located on chromosome 7 (Table 3). The candidate genes containing these SNP markers and their putative functions were identified through BLAST searching the sorghum v3.1 genome at Phytozome 12.1 (Table 3).

**TABLE 3:** The list of 13 SNP loci that were identified as loci under selection and their descriptions.

SNP markers	Chr.	SNP Pos.	Ref/Alt	MAF	Het	F_ST_	Map pos	Candidate Gene	Annotation
snp_sb001000600993	6	4719375	T/A	0.33	0.30	0.91	4719016.4719660	Sobic.006G026400	No annotated domain for this protein
snp_sb001000665443	6	30979222	G/A	0.34	0.27	0.89	30971365.30982488	Sobic.006G044400	Similar to OSIGBa0097I24.1 protein
snp_sb042060260697	1	67217203	T/C	0.17	0.46	0.83	67215996.67219771	Sobic.001G384700	Similar to Zinc finger C-x8-C-x5-C-x3-H type familyprotein, expressed
snp_sb042060594735	5	61927575	C/T	0.29	0.35	0.94	61927471.61932993	Sobic.005G150200	Weakly similar to Putative uncharacterized protein
snp_sb042060764024	7	5101345	A/G	0.18	0.45	0.84	5098301.5104103	Sobic.007G050400	Similar to DEAD-box ATP-dependent RNA helicase 42
snp_sb042060834108	7	54216799	C/A	0.34	0.28	0.88	54216242.54222782	Sobic.007G127600	Similar to Os02g0653400 protein
snp_sb042060834531	7	54578827	G/C	0.23	0.41	0.84	54578165.54580032	Sobic.007G129300	Weakly s.t Putative uncharacterized protein
snp_sb042060855091	7	65318268	C/G	0.13	0.49	0.91	65311844.65318939	Sobic.007G225800	Similar to Proliferating-cell nucleolar antigen-like protein
snp_sb042060855137	7	65355302	A/C	0.13	0.49	0.91	65354308.65356452	Sobic.007G226300	Similar to Putative uncharacterized protein
snp_sb042060855138	7	65357757	T/C	0.13	0.49	0.91	65356668.65359547	Sobic.007G226400	Similar to Pentatricopeptide (PPR) repeat-containing protein-like
snp_sb042060855361	7	65431483	C/T	0.13	0.49	0.91	65424095.65433354	Sobic.007G227100	Similar to Os08g0482100 protein
snp_sb042061021321	9	52527630	C/T	0.14	0.48	0.83	52527443.52529178	Sobic.009G169300	Weakly similar to Os05g0470900 protein
snp_sb042061023058	9	53254749	T/A	0.05	0.51	0.81	53254243.53259288	Sobic.009G177600	K10683 - BRCA1-associated RING domain protein 1

*Chr., chromosome; SNP Pos., SNP position in the corresponding sorghum chromosome; Ref/Alt, reference and alternative alleles; MAF, minor allelic frequency; Het, heterozygosity; F_ST_, population differentiation; Map Pos., map positions of the candidate genes in the corresponding sorghum chromosome.*

### Cluster Analyses of Individual Genotypes and Accessions

**FIGURE 7:**
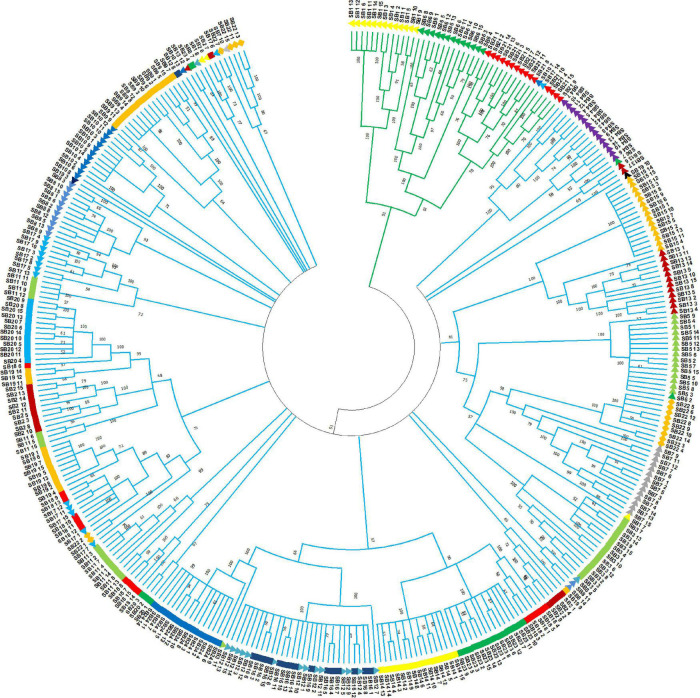
Unweighted pair group method with arithmetic mean (UPGMA) dendrogram of 359 individuals representing the 24 sorghum accessions generated based on Nei’s genetic distance (Nei and Takezaki, 1983). The individual samples were coded in a way that the first two letters (SB) with either two- or three-digit numbers represent their accessions and the last two-digit numbers represent the codes for the individual plant in that accession. Individuals denoted by the same color and shape belong to the same accession.

**FIGURE 8:**
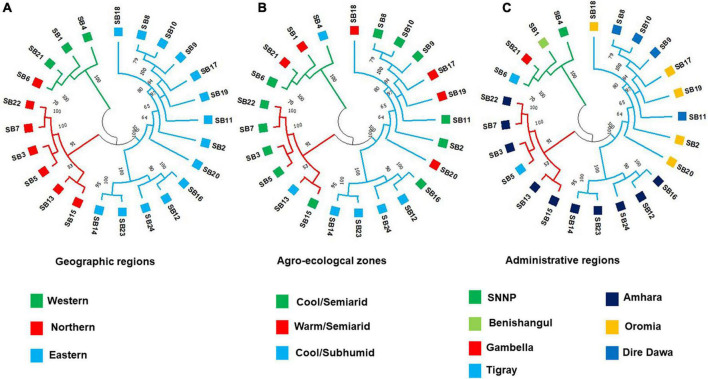
The UPGMA dendrogram of the 24 accessions generated based on Nei’s genetic distance (Nei and Takezaki, 1983) calculated using the genotypic data of 3,001 SNP markers. Accessions denoted by the same color labels belong to the same **(A)** geographic region, **(B)** agro-ecological zone, and **(C)** administrative region.

The unweighted pair group method with arithmetic mean-based cluster analysis of the 359 individual genotypes generated a dendrogram of three major clusters, which were denoted by different line colors in Figure 7. The cluster analysis at the accession level resulted in the clustering of the 24 accessions into two groups. In the case of individual genotypes, Cluster I consisted of 316 individuals, whereas Cluster II comprised 43 individuals, respectively. The cluster analysis showed that at least the majority of individuals from the same accessions were clustered together (Figure 7). All individuals of an accession were clustered closely together in several cases. For example, all individuals from accessions, *SB21* and *SB4* were clustered in Cluster I and Cluster II, respectively. In other cases, a few individuals of an accession were placed under different clusters. For instance, two individuals from accession *SB1*, one individual from *SB6*, *SB17*, and *SB22* were separated from the other members of their accession and grouped with other genotypes in different clusters. Except in a few cases, most accessions were clearly clustered based on their geographic regions (Figure 8A). On the other hand, the clustering pattern of the accessions according to their agro-ecological zones or administrative regions was less resolved, as accessions were mostly clustered irrespective of their groups (Figures 8B,C).

### Principal Coordinate Analysis

**FIGURE 9:**
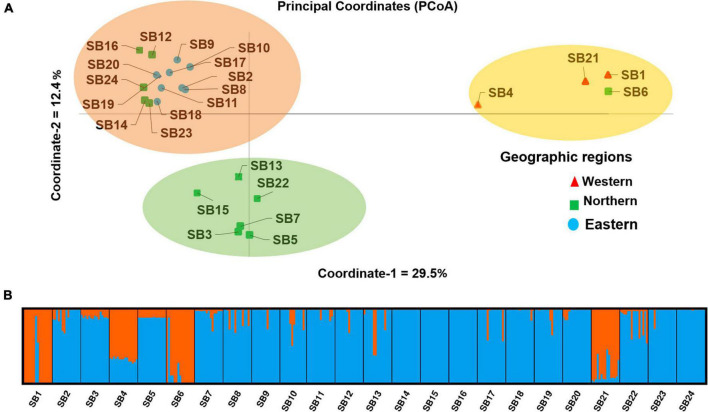
**(A)** Principal coordinates analysis (PCoA) showing the clustering pattern of the 24 Ethiopian sorghum accessions and accessions denoted by the same color labels and shapes belonging to the same geographic region and **(B)** a graphical display of the population genetic structure of the 24 sorghum accessions for K = 2. The two colors represent the two clusters (genetic populations) and each color of an accession represents the average proportion of the alleles that placed that accession under the corresponding clusters.

Principal coordinate analysis was performed to determine the relationship between the sorghum accessions and individuals within the accession, which grouped the accessions into three separate clusters (Figure 9A and Supplementary Figure 4). The first and second coordinates explained 29.5 and 12.4% of the total variation among the accessions, respectively. Similar to the cluster analysis, PCoA revealed that accessions *SB1*, *SB4*, *SB6*, and *SB21* are the most differentiated groups being clearly separated from the other accessions along the first principal coordinate (Figure 9A).

### Population Structure

The admixture model-based population structure of the 359 individuals representing the 24 accessions was inferred using STRUCTURE software. The analysis of the STRUCTURE output using STRUCTURE HARVESTER program (Earl, 2012) that implemented ΔK method of Evanno et al. (2005) revealed that the optimal number of genetic clusters is two (Supplementary Figure 5). The results suggest that the 24 sorghum accessions originate from two genetic populations as graphically depicted in Figure 9B. In line with the results of the cluster analysis and PCoA, accessions *SB1, SB4, SB6*, and SB21 were significantly differentiated groups, as the majority of their alleles belong to a different genetic population (represented by orange in Figure 9B) as compared to the other accessions.

## Discussion

### SNP Markers and Their Use in Genetic Diversity Analysis of Sorghum Gene Pool

Genetic diversity analysis of crop species is an important step in detecting alleles that could be used for their improvement through breeding. The Ethiopian sorghum gene pool has been used as a novel source of biotic and abiotic stress tolerance, greatly contributing to the improvement of sorghum, globally (Adugna, 2014). The gene pool has been utilized in various studies that aimed at the identification of novel QTLs and genes governing complex traits (Cuevas and Prom, 2013, 2020; Cuevas et al., 2017; Menamo et al., 2021). Since polymorphism within genes or their close vicinity is expected to be the main basis of phenotypic variation, priority was given to SNPs located in genes in the SNP selection process in this study. Because of simplicity and abundance in plant genomes, bi-allelic SNPs are the most commonly used SNPs used in genetic analyses. In the present study, 86% of the genotyped bi-allelic SNP loci were polymorphic, which can be considered high. This is most likely because, the SNP selection was mainly made based on the SNPs recorded for the Ethiopian sorghum genotype, *Cherekit* at the SorGSD database. The vast majority of the SNP loci (94.1%) were homozygous across the 359 individual samples genotyped, which is not surprising as sorghum is a self-pollinating crop.

The variation in allele frequency distribution among accessions shown by the analysis of site frequency spectrum indicates a high level of genetic diversity in the Ethiopian sorghum. Accessions containing individual genotypes dominated by minor alleles across the loci require further investigations to reveal the phenotypic diversity of desirable traits. The overall nucleotide diversity (π) of 1.2 recorded in this study is in agreement with the result of a previous study on sorghum landraces (Mace et al., 2013). However, it is higher than the values reported in some other studies on sorghum (Morris et al., 2013; Yan et al., 2018). Similarly, the overall Tajima’s D value recorded in this study was 1.4, which is lower and higher than values reported in Morris et al. (2013) and (Mace et al., 2013), respectively. Among the seven starch-related genes, *amylose extender1* (*ae1*), *brittle2* (*bt2*), *Opaque2* (*O2*), *shrunken1* (*sh1*), *shrunken2* (*sh2*), *sugary1* (*su1*), and w*axy1* (*wx1*), previously identified as candidates of domestication loci (Whitt et al., 2002; De Alencar Figueiredo et al., 2010; Morris et al., 2013), three of them (*sh2*, *ae1*, and *bt2*) are found at the genomic regions with notably low diversity on chromosomes 2, 4, and 7, respectively (Figure 2). The *bt2* gene on chromosome 7 coding for a starch biosynthesis enzyme has been shown to be a likely domestication locus in sorghum and maize (Whitt et al., 2002; De Alencar Figueiredo et al., 2010; Morris et al., 2013). The low recombination rates in the pericentromeric region or the presence of other loci under selection in this region may be the reason for the low diversity in the present study and previous studies on sorghum (Morris et al., 2013).

The average Ho of 0.06 obtained in the present study was in line with the results of previous studies on sorghum employing SNP markers (Cuevas et al., 2017) and SSR markers (Ng’uni et al., 2011) (Ho = 0.04), (Ramu et al., 2013; Motlhaodi et al., 2014) (Ho = 0.09), (Motlhaodi et al., 2017) (Ho = 0.03). The Ho was expected, as sorghum is a predominantly a self-pollinating crop (Poehlman and Sleper, 1979). Gene diversity (H) and PIC are the most common measures of polymorphism of markers, which shed light on the evolutionary pressure on the alleles and the mutation rate at a locus over time (Shete et al., 2000; Wilkinson et al., 2012). The total genetic diversity in a population can be estimated through the analyses of a large number of informative markers across their genome (Melchiorre et al., 2013). The gene diversity of the SNP markers across all accessions in this study ranged from 0.1 to 0.50 with a mean of 0.29, which is high. Informative markers could be used for genotyping populations for genetic diversity studies, and the informativeness of the markers can be measured by their PIC value (Salem and Sallam, 2016). In the case of bi-allelic SNP markers, the maximum PIC value of 0.375 is attained when both alleles have a frequency of 0.5. In the present bi-allelic SNP-based study, the PIC values ranged from 0.09 to 0.375 with the overall average of 0.24, which is comparable with previous studies on sorghum using SNP markers (Afolayan et al., 2019; Silva et al., 2021; Wondimu et al., 2021). Forty-seven percent of these SNP loci have a PIC value of greater than 0.25 and hence they are highly informative and could be used for various applications including population genetic studies of sorghum.

Selections, both natural and artificial, as well as inbreeding, contribute to the deviation of populations from HWE. In this study, 98% of the loci showed heterozygote deficiency while 1.60% of the loci showed excess heterozygosity. Since sorghum is a predominately self-pollinating species, heterozygote deficiency at the vast majority of the loci can be attributed to inbreeding. However, the small proportion of loci showing excess heterozygosity suggests that they could be under selection or linked to loci under selection. Among the SNP loci that showed excess heterozygosity, nine loci lacked one of the two homozygous genotypes. The data suggest that one of the two alleles at each locus reduces the fitness of homozygous genotypes, or the locus is linked to another locus within its corresponding gene or the nearby gene that has a significant fitness value. Most of these SNP markers are within the coding region of genes. For instance, snp_sb001000687053, snp_sb001000723312, snp_sb042060543510, snp_sb042060515233, and snp_sb042060517985 are within the coding region of senescence-related gene 1, tetratricopeptide repeat (TPR)-like superfamily protein, cysteine proteinases superfamily protein, C-terminal domain phosphatase-like 4, and hydroxyproline-rich glycoprotein family protein, respectively. These genes had major roles in the growth, development, physiology, and biotic and abiotic stress tolerances in plants. For instance, hydroxyproline-rich glycoproteins (HRGPs) play a major role in the growth and development of plants (Showalter et al., 2016) while cysteine proteinases play an important physiological process ranging from seed germination (Becker et al., 1994) to senescence (Valpuesta et al., 1995). Therefore, further study that investigates the effect of these SNPs on the response of sorghum to abiotic and biotic stresses is of high significance.

### Genetic Diversity Within Accessions

The average He (0.15), I (0.25), and PPL (47.7%) obtained in the present study suggest low genetic variation within the sorghum accessions. In general, the relatively low genetic variation within landrace accessions in the present and previous studies on sorghum (Ng’uni et al., 2011; Motlhaodi et al., 2017) is likely due to the combination of its inbreeding nature and due to the strict selection criteria of farmers. However, the variation within accessions varied widely. In this regard, accessions from the western region [Benishangul-Gumuz, Gambella, and Southern Nations, Nationalities and Peoples’ Region (SNNPR)] had higher variation than other accessions with *SB21* being the most diverse accession followed by *SB4*. Accessions from this region (*SB21, SB1*, and *SB4*) are characterized by bent peduncle and light brown seeds with the exception of the red seed color of *SB1*.

Mengistu et al. (2020) also reported higher gene diversity and PIC for accessions from the Benshangul-Gumuz, Gambella, and SNNP regions as compared to the other regions in Ethiopia. The higher variation within accessions from these regions may suggest less human selection pressure on the landraces as compared to sorghum grown elsewhere in the country. Since the genetic diversity of populations implies their potential to adapt to environmental changes (Markert et al., 2010), sorghum landraces from this region may serve as a potential source of genes for biotic and abiotic stresses. Another interesting result of this study is a significantly higher Ho in two of the three accessions (*SB4* and *SB21*) from the western regions as compared to all other accessions. Higher Ho suggests a higher outcrossing rate in these accessions, which might have allowed for gene flow through pollen and hence increased the variation within the accessions. The results suggest the western region as an important source of sorghum genotypes with desirable traits, such as tolerance to biotic and abiotic stresses. On the other hand, most accessions from Northern Ethiopia (Tigray and Amhara) had very low variation within accessions. Their average Ho was 0.03, indicating that the vast majority of the loci in the genotypes of these accessions were homozygous. In this group, accessions *SB5*, *SB14*, *SB15*, and *SB16* can be regarded as pure lines, as individuals within each accession are almost identical across the whole loci. On the other hand, other accessions in this group (*SB6*, *SB13*, and *SB22*) are more diverse although their heterozygosity is still very low. Since the loss of heterozygosity increases the chance of deleterious recessive alleles being expressed in the progeny (Radosavljević et al., 2015), these accessions may be more susceptible to biotic or abiotic stresses unless they have been selected for tolerance against these stresses over time.

### Genetic Differentiation of Accessions and Hierarchical Groups

In this study, most of the total variations (64.5%) were observed among the accessions than within the accessions (35.5%). The lower genetic variation within the accessions is expected in self-pollinating crops like sorghum (Hamrick, 1983). In addition, strict farmers’ selection for crop improvement might have contributed to the lower within-accession variation, which were clearly displayed in accessions, such as *SB14* and *SB15*. Previous genetic diversity studies through SNP and SSR markers also showed a higher genetic variation among sorghum accessions than within the accessions. For instance, SNP-based genetic diversity study on sorghum accessions from Ethiopia showed that the variation among and within the accessions accounted for 59.6 and 40.4% of the total variation, respectively (Mengistu et al., 2020). Similarly, genetic diversity study through SSR markers on sorghum accessions from Zambia revealed 82 and 18% genetic variations among and within the accession variations, respectively (Ng’uni et al., 2011). Motlhaodi et al. (2017) reported a significant genetic variation among 22 accessions of sorghum, which accounted for 66.9% of the total variation while the within accession variation accounted for 23.6%. However, high genetic variation within sorghum accessions were reported on sorghum studied through SNP markers (Afolayan et al., 2019) and SSR markers (Manzelli et al., 2007; Adugna, 2014), suggesting that the accessions are not under selection processes.

Several studies have shown that the diversity of sorghum is associated with geography, agro-ecology, ethnicity, or botanical racial classifications (Barnaud et al., 2007; Ng’uni et al., 2011; Faye et al., 2019; Menamo et al., 2021). Significant genetic variations among the geographic regions and peduncle shape groups were observed in this study as shown by hierarchical AMOVA. Among the geographic regions, the western and eastern regions had higher genetic diversity than the northern region as shown by average He and the percentage of polymorphic loci, which were higher than the overall average (He = 0.24 and PIC = 89%). The western region accessions were the most distinct, with higher differentiation from those of the northern and eastern geographic regions. The major sorghum growing area (northern region) of the country had relatively low genetic variation probably due to intensive farmers’ selection of landraces to cope with the local environmental factors, such as the duration of the rainy season. The diversity of the crop has been reduced over time due to the recurrent drought in this major sorghum-growing region of the country. Overall, farmers in the drought-prone lowland areas tend to use early maturing and high yielding types and or shift their production systems to more vulnerable and low yielding early maturing crop species, such as tef (*Eragrostis tef*) (Adugna, 2014), which may provide genetic erosion of the sorghum landraces in these regions. High adoption of early maturing improved varieties in drought-prone areas in the northern region was also reported (Tesfaye et al., 2013).

Private alleles represent a unique genetic variability at certain loci of a particular population or hierarchically grouped populations. In this study, private alleles were not detected at the population level, but were recorded in all geographic regions. The Eastern region had a higher number of private alleles as compared to the western and northern regions, and hence it may serve as a rich source of desirable alleles for sorghum improvement. Private alleles generally support the potential to respond to a selection or have evolutionary significance (Petit et al., 1998). Information on private alleles is crucial for selecting highly diverse genotypes that can be used in breeding programs as a source of parental lines for crossbreeding that would eventually lead to new cultivars enriched with desirable alleles (Brondani et al., 2006; De Oliveira Borba et al., 2009; Salem and Sallam, 2016). The presence of more private alleles in the eastern region suggests the good *in situ* conservation status of sorghum in that location. Hence, further studies that explore the region for highly desirable traits need to be conducted, especially for use in sorghum-breeding programs.

Sorghum genotypes showed a significant genetic differentiation based on their peduncle shape, possibly because the shape of the peduncle influences the mating system, with the architecture of very bent peduncle obstructing pollination with outcrossed pollen. A more interesting finding was that accessions with bent peduncles exhibited higher genetic variation on average than those with erect peduncles. Unlike previous studies on Ethiopian sorghum (Menamo et al., 2021; Wondimu et al., 2021), sorghum accessions were not significantly differentiated according to agro-ecology in this study. However, a high significant genetic difference among the three pairs of agro-ecological zones was observed and the warm/semiarid zones showed the highest genetic diversity among the agro-ecological zones. Private alleles were detected from warm/semiarid and cool semiarid zones. Cool/subhumid zones, however, did not exhibit any private allele.

In the present study, 13 SNP loci were identified as loci under selection through the determination of the joint distribution of F_ST_ and heterozygosity. More than 50% of these SNP loci are located on chromosome 7 of the sorghum genome, suggesting that this chromosome carries many genes under natural selection or targeted by farmers directly or indirectly during and after domestication. These SNPs include those located in genes coding for zinc finger CCCH type family protein, DEAD-box ATP-dependent RNA helicase 42 and pentatricopeptide (PPR) repeat-containing proteins, which play a crucial role in plant responses to biotic and abiotic stresses (Peng et al., 2012; Xing et al., 2018; Nidumukkala et al., 2019). Hence, further study on these loci using individual genotypes that carry different alleles may shed more light on their significance in terms of desirable traits.

### The Clustering Pattern and Population Structure of the Sorghum Accessions

Unweighted pair group method with arithmetic mean clustering based on Nei’s genetic distance (Nei and Takezaki, 1983) placed the individuals from the 24 accessions into three clusters. In line with the generally low genetic variation within accessions revealed through different analyses, there was a clear clustering pattern of individual genotypes according to their accessions. At the accession level, the cluster analysis generated three distinct clusters that matched the three clusters of the PCoA, which explained 42% of the total variation in its first two principal axes. The STRUCTURE analysis also generally agrees with the observed clustering pattern although it suggested two genetic populations (K = 2) as the best representation of the germplasm studied. Most of the alleles of the most distinct clusters in UPGMA and PCoA analyses (containing *SB1*, *SB4*, *SB6*, and *SB21*) originate from the first genetic cluster of STRUCTURE analysis (shown orange in Figure 9). Hence, it is interesting to crossbreed individual genotypes in these accessions with genotypes of genetically uniform accessions (e.g., *SB14* and *SB15*), and evaluate the progeny generations for desirable traits.

In this study, the significant differentiation among geographic groups but not among agro-ecological groups revealed through AMOVA was also evident in the cluster analysis at the level of accessions. Based on redundancy analysis in their recent study on sorghum, Menamo et al. (2021) reported that agro-ecology is more important than the administrative region in defining the genetic variation in sorghum, which is not in agreement with the present study. The present study showed that the genetic diversity of Ethiopian sorghum landrace accessions was more structured along the geographical regions than along the administrative regions or agro-ecological zones. The lack of clear genetic differentiation of sorghum along the administrative regions, which was also previously reported (Ayana and Bekele, 2000; Desmae et al., 2016; Wondimu et al., 2021), could be explained by a high gene flow because of extensive exchange of seeds among farmers across adjacent regions where sorghum is a major crop.

## Conclusion

In this study, SeqSNP method was used to genotype diverse sorghum accessions using a combination of previously developed and newly identified gene-based SNP markers. Despite the fact that they were gene-based, the SNP markers revealed a comparable genetic variation from the previous studies using SNP markers in sorghum. About half of the SNP markers can be regarded as highly informative and can be prioritized for future population genetics studies. A significant number of loci exhibited excess heterozygosity and/or were presumed to be under selection, some of which are located within genes playing crucial roles in plant responses to biotic and abiotic stresses. Further research on these loci using genotypes carrying different alleles may shed light on their significance in terms of desirable traits. The observed highly significant genetic differentiation among the sorghum accessions will be beneficial to the sorghum breeders in selecting desirable parents for crossbreeding. The sorghum accessions formed three distinct clusters, and it is therefore interesting to crossbreed genotypes from different clusters to evaluate their progeny for desirable traits. In this study, highly significant variations were observed among the geographic regions and peduncle-shaped groups. Compared to the western and northern regions, the eastern region had a higher number of private alleles, and hence it may serve as a rich source of desirable alleles for improving sorghum. Lastly, given that sorghum is generally regarded as a self-pollinating species, an exceptionally high heterozygosity observed in accessions, namely, *SB4* and *SB21* from the western geographic region, is an interesting result of this study, and should be further investigated.

## Data Availability Statement

The datasets presented in this study can be found in online repositories. The names of the repository/repositories and accession number(s) can be found in the article/Supplementary Material.

## Author Contributions

MG and ME designed the experiment and analyzed the data. ME conducted the experiment and wrote the draft manuscript. AC, CH, KT, MG, and TF reviewed the manuscript. All authors conceived the study and read and approved the submission of the manuscript for publication.

## Conflict of Interest

The authors declare that the research was conducted in the absence of any commercial or financial relationships that could be construed as a potential conflict of interest.

## Publisher’s Note

All claims expressed in this article are solely those of the authors and do not necessarily represent those of their affiliated organizations, or those of the publisher, the editors and the reviewers. Any product that may be evaluated in this article, or claim that may be made by its manufacturer, is not guaranteed or endorsed by the publisher.

## Footnotes

1
http://genome.jgi-psf.org/Sorbi1/Sorbi1.info.html

2
https://www.ebi.gov.et/biodiversity/conservation/genetic-material-holdings/

3
https://biosearch-cdn.azureedge.net/assetsv6/sbeadex-plant-data-sheet.pdf

4
http://sorgsd.big.ac.cn

5
www.phytozome.net

6
https://ngdc.cncb.ac.cn/sorgsd/

## Supplementary Material

Supplementary Figure 1 Geographical maps of Ethiopia showing **(A)** the original sampling locations of the sorghum accessions with red, green, and blue colors to highlight the western, northern, and eastern geographic regions, respectively and **(B)** the agro-ecological zones of Ethiopia as per the Global 16 Class classification system by Amede et al. (2015).

Supplementary Figure 2 The panicle diversity of sorghum landraces grown in the country.

Supplementary Figure 3 Minor allelic frequency (MAF) range of 3,001 SNP markers used for genetic diversity and population structure analyses of 359 individual plants representing the 24 sorghum accessions.

Supplementary Figure 4 Principal coordinates analysis (PCoA) showing the clustering pattern of the 359 individuals of sorghum landraces and individuals denoted by the same color labels and shapes belonging to the same geographic region.

Supplementary Figure 5 Inferred population structure of 24 sorghum accessions at K = 2. ΔK plot showing its maximum value at K = 2 suggesting two as the optimal number of genetic populations.
