# Supplementary figures and images for: Genetic Diversity and Population Structure of Sorghum [Sorghum Bicolor (L.) Moench] Accessions as Revealed by Single Nucleotide Polymorphism Markers
Source: Front Plant Sci. 2022 Jan 5;12:799482. doi: 10.3389/fpls.2021.799482 (PMC8766336; doi:10.3389/fpls.2021.799482)

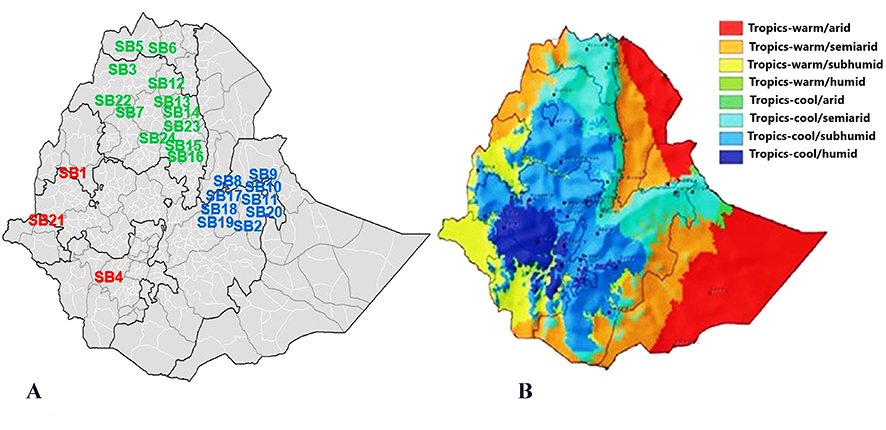

Supplement: Supplementary Figure 1 — Geographical maps of Ethiopia showing (A) the original sampling locations of the sorghum accessions with red, green, and blue colors to highlight the western, northern, and eastern geographic regions, respectively and (B) the agro-ecological zones of Ethiopia as per the Global 16 Class classification system by Amede et al. (2015). [file Image_1.TIF]

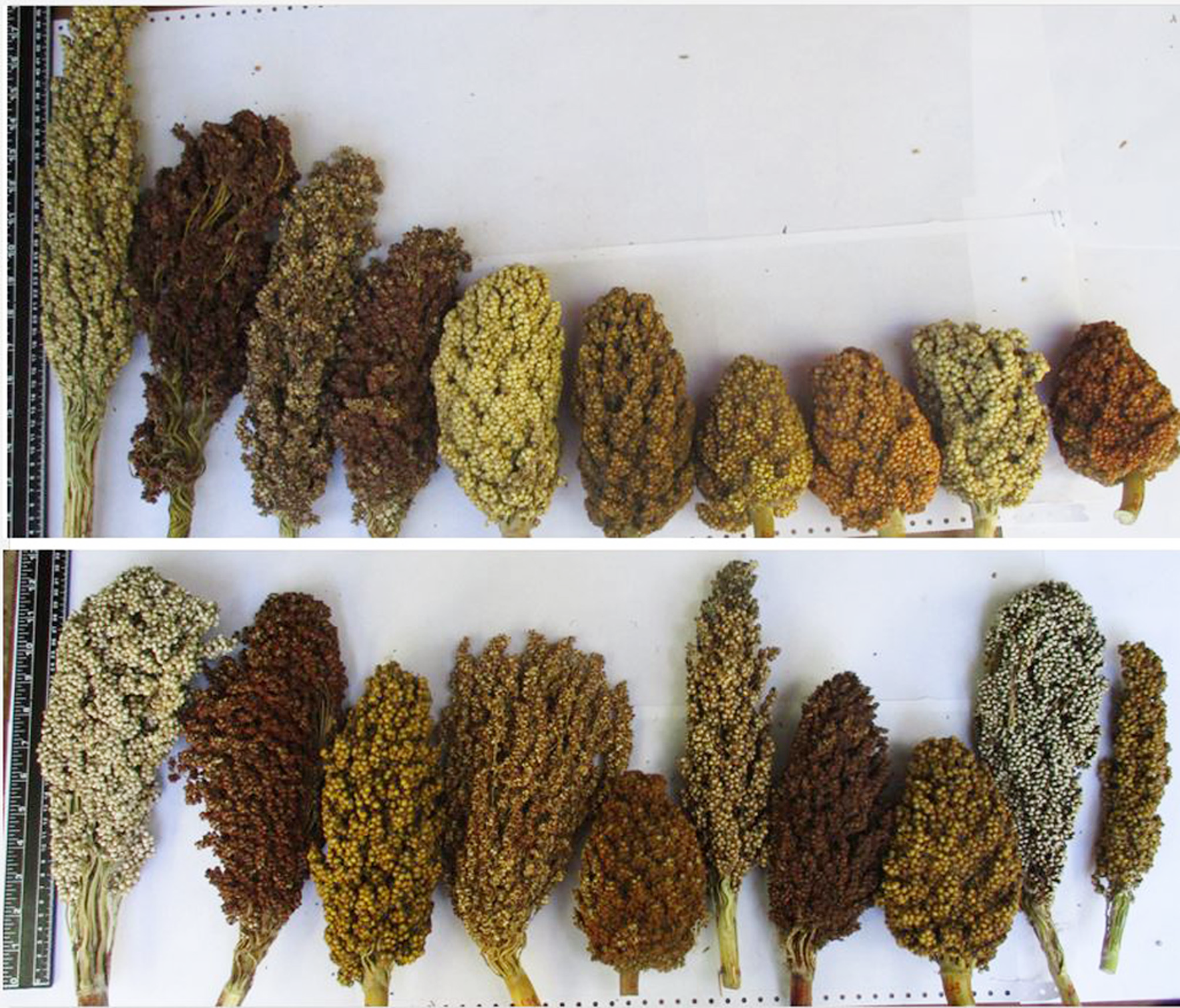

Supplement: Supplementary Figure 2 — The panicle diversity of sorghum landraces grown in the country. [file Image_2.TIFF]

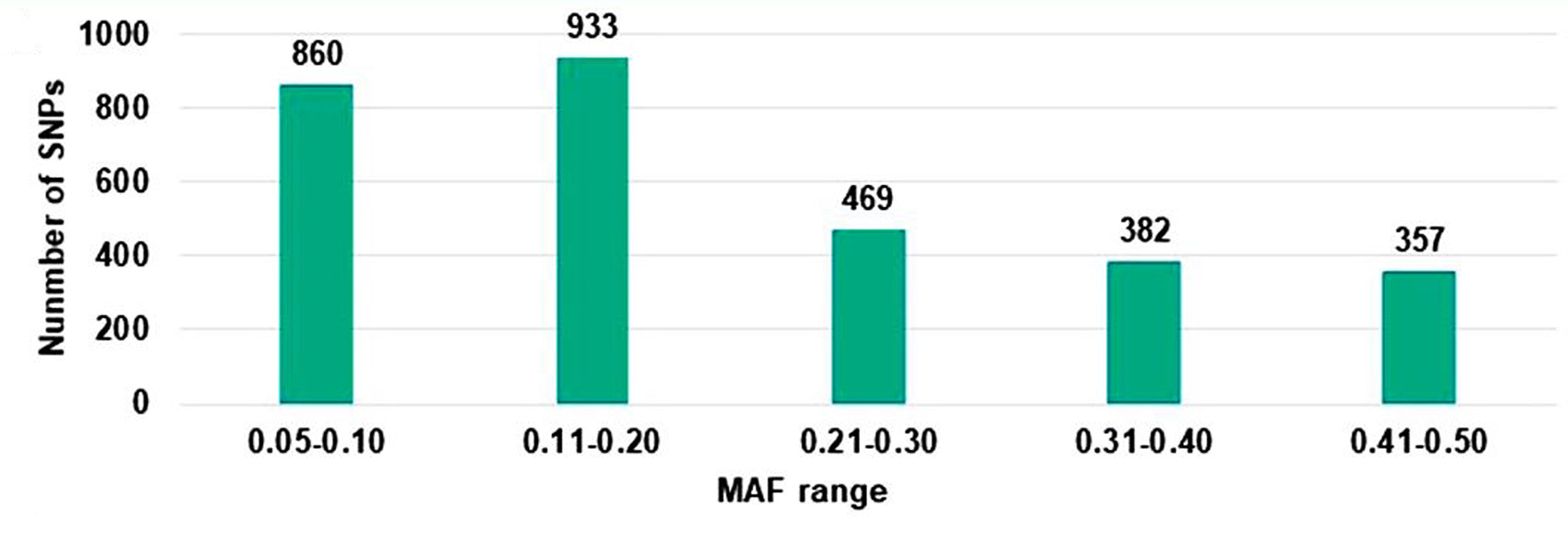

Supplement: Supplementary Figure 3 — Minor allelic frequency (MAF) range of 3,001 SNP markers used for genetic diversity and population structure analyses of 359 individual plants representing the 24 sorghum accessions. [file Image_3.TIF]

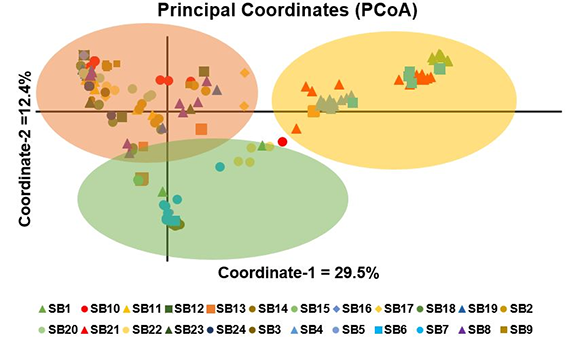

Supplement: Supplementary Figure 4 — Principal coordinates analysis (PCoA) showing the clustering pattern of the 359 individuals of sorghum landraces and individuals denoted by the same color labels and shapes belonging to the same geographic region. [file Image_4.TIFF]

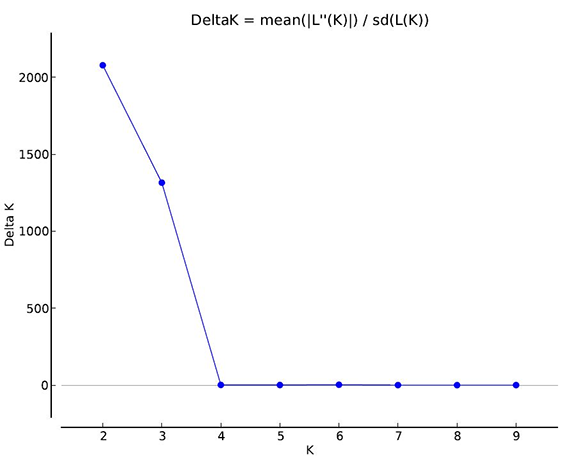

Supplement: Supplementary Figure 5 — Inferred population structure of 24 sorghum accessions at K = 2. ΔK plot showing its maximum value at K = 2 suggesting two as the optimal number of genetic populations. [file Image_5.TIFF]
